# The Glasgow Microenvironment Score associates with prognosis and adjuvant chemotherapy response in colorectal cancer

**DOI:** 10.1038/s41416-020-01168-x

**Published:** 2020-11-23

**Authors:** Peter G. Alexander, Antonia K. Roseweir, Kathryn A. F. Pennel, Hester C. van Wyk, Arfon G. M. T. Powell, Donald C. McMillan, Paul G. Horgan, Caroline Kelly, Jennifer Hay, Owen Sansom, Andrea Harkin, Campbell S. D. Roxburgh, Janet Graham, David N. Church, Ian Tomlinson, Mark Saunders, Tim J. Iveson, Joanne Edwards, James H. Park

**Affiliations:** 1 School of Medicine, University of Glasgow, Glasgow, UK; 2 Institute of Cancer Sciences, University of Glasgow, Glasgow, UK; 3 Division of Cancer and Genetics, Cardiff University, Cardiff, UK; 4 CRUK Clinical Trials Unit, The Beatson West of Scotland Cancer Centre, Gartnavel Hospital, Glasgow, UK; 5 Glasgow Tissue Research Facility, University of Glasgow, Queen Elizabeth University Hospital, Glasgow, UK; 6 CRUK Beatson Institute of Cancer Research, Garscube Estate, Glasgow, UK; 7 Wellcome Centre for Human Genetics, University of Oxford, Oxford, UK; 8 NIHR Oxford Biomedical Research Centre, Oxford University Hospitals NHS Foundation Trust, John Radcliffe Hospital, Oxford, UK; 9 Edinburgh Cancer Research Centre, IGMM, University of Edinburgh, Crewe Road, Edinburgh, EH4 2XU UK; 10 The Christie NHS Foundation Trust, Manchester, UK; 11 Southampton University Hospital NHS Foundation Trust, Southampton, UK

**Keywords:** Prognostic markers, Predictive markers, Outcomes research, Surgical oncology

## Abstract

**Background:**

The Glasgow Microenvironment Score (GMS) combines peritumoural inflammation and tumour stroma percentage to assess interactions between tumour and microenvironment. This was previously demonstrated to associate with colorectal cancer (CRC) prognosis, and now requires validation and assessment of interactions with adjuvant therapy.

**Methods:**

Two cohorts were utilised; 862 TNM I–III CRC validation cohort, and 2912 TNM II–III CRC adjuvant chemotherapy cohort (TransSCOT). Primary endpoints were disease-free survival (DFS) and relapse-free survival (RFS). Exploratory endpoint was adjuvant chemotherapy interaction.

**Results:**

GMS independently associated with DFS (*p* = 0.001) and RFS (*p* < 0.001). GMS significantly stratified RFS for both low risk (GMS 0 v GMS 2: HR 3.24 95% CI 1.85–5.68, *p* < 0.001) and high-risk disease (GMS 0 v GMS 2: HR 2.18 95% CI 1.39–3.41, *p* = 0.001). In TransSCOT, chemotherapy type (*p*_interaction_ = 0.013), but not duration (*p* = 0.64) was dependent on GMS. Furthermore, GMS 0 significantly associated with improved DFS in patients receiving FOLFOX compared with CAPOX (HR 2.23 95% CI 1.19–4.16, *p* = 0.012).

**Conclusions:**

This study validates the GMS as a prognostic tool for patients with stage I–III colorectal cancer, independent of TNM, with the ability to stratify both low- and high-risk disease. Furthermore, GMS 0 could be employed to identify a subset of patients that benefit from FOLFOX over CAPOX.

## Background

Colorectal cancer (CRC) poses a significant burden on healthcare worldwide, with 1.8 million CRC-related deaths in 2018.^1^ TNM staging remains the primary tool for guiding prognosis and management following CRC resection.^2,3^ However, there are wide variations in prognosis for individuals within the same TNM stage.^4^ Further, high-risk features have been identified for stage II disease selecting patients for consideration of adjuvant therapy.^5,6^ However, Dienstmann et al.,^4^ when analysing the relative impact of TNM, clinicopathological features and molecular markers on survival outcomes, reported that the additional features to the TNM only modestly increased prognostic accuracy.

Clearly, further prognostic markers are required and the interaction between host and tumour is integral to this process. Two independent prognostic scoring systems assessing the tumour microenvironment have been developed, namely tumour stromal percentage (TSP) and an assessment of peritumoural inflammation, both of which remain optional in the current edition of the Royal College of Pathologists colorectal cancer reporting dataset.^3^ As the local inflammatory response is fundamental in orchestrating host antitumour immunity,^7^ an increase in infiltrating immune cell density is recognised as a stage-independent favourable prognostic characteristic^8,9^ and a recent study in colon cancer highlighted tumour immunity as pivotal to accurate assessment of recurrence risk in conjunction with TNM.^10^ Similarly, higher TSP is a validated, poor prognostic marker independent of TNM in CRC^11^ and has more recently been associated with the mesenchymal consensus molecular subtype.^12^

Assessment of the inflammatory cell infiltrate and mesenchymal phenotype retain independent and complementary prognostic value in patients with operable CRC, and several groups have proposed their combined assessment as an adjunct to staging.^13–15^ The Glasgow Microenvironment Score (GMS) combines assessment of peritumoural inflammation, using the Klintrup–Mäkinen grade (KM), with assessment of TSP, both performed on routinely available haematoxylin and eosin (H&E)-stained sections.^11,16,17^ Its clinical utility has been reported in a discovery cohort, stratifying 5-year cancer-specific survival of 307 patients with stage I–III CRC into three distinct groups.^13^

The GMS now requires validation and could provide a platform on which to develop personalised treatment approaches for CRC, which is also important for adjuvant chemotherapy, where biomarkers are lacking. For example, the SCOT trial recently demonstrated patients receiving CAPOX (capecitabine and oxaliplatin) have similar survival with 3- versus 6-months duration, whereas patients receiving FOLFOX (bolus and infused fluorouracil with oxaliplatin) may benefit from 6-months duration.^18,19^ Therefore, it is important to identify patients who may benefit from a longer and more intensive chemotherapy regimen. Recently we investigated the utility of a histopathology-based classification of the Consensus Molecular Subtypes called Phenotypic Subtypes, incorporating KM grade, TSP and the proliferation marker Ki67.^20^ This stratified chemotherapy response in a cohort of 1343 patients from the adjuvant chemotherapy SCOT trial (TransSCOT), with the predictive power of this subtyping predominantly related to assessment of KM grade and TSP. Therefore, it was deemed more appropriate and pragmatic in the current study to use GMS to assess the expanded cohort in preference to Phenotypic Subtypes, since the GMS can be performed on H&E slides that are routinely used in histopathological staining without the need for immunohistochemistry.

Therefore, the primary aim of the present study was to assess the validity of the GMS as a prognostic score in two independent cohorts: an expanded validation cohort of stage I–III CRC patients and the full TransSCOT cohort. The exploratory aim was to assess associations of GMS with adjuvant chemotherapy type and duration in the TransSCOT cohort.

## Methods

### Patients

The validation cohort included 862 TNM I–III CRC, combining individuals from the discovery Glasgow Royal Infirmary cohort(*n* = 231) with additional patients identified retrospectively from other Glasgow hospitals (Western Infirmary, Gartnavel General and Stobhill Hospitals) who had undergone surgery with curative intent from 2000–2007 (*n* = 631). Patients undergoing palliative or endoscopic resections and those with involved surgical margins (R1 resections) were excluded. In the TransSCOT cohort there were 2912 patients with available tissue from the SCOT adjuvant chemotherapy trial (ISRCTN no. 59757862) who had undergone potentially curative resection for high-risk TNM II or TNM III CRC from 2008–2013 within the UK. All patients were followed up for at least 3 years. 30-day surgical mortalities were excluded from both cohorts.

### Study endpoints

The primary endpoint was disease-free survival (DFS; measured from date of surgery/randomisation to date of recurrence or all-cause mortality) for both the validation and TransSCOT cohorts. In addition, relapse-free survival (RFS; measured from date of surgery to date of recurrence or CRC-related mortality), cancer-specific survival (CSS; measured from date of surgery until CRC-related mortality) and overall survival (OS; measured from date of surgery until all-cause mortality) were calculated for the validation cohort. Furthermore, the interaction between GMS, adjuvant chemotherapy type and duration and DFS was examined in the TransSCOT cohort. Survival data for the validation cohort was complete up until 9 February 2017, which acted as censor date, and until end of study period for the TransSCOT cohort.

### Clinicopathological characteristics

#### Validation cohort

Clinical characteristics were recorded from patient case notes, and pathological characteristics, including TNM, were collected from pathology reports. Venous invasion was assessed using H&E-stained sections (both intra- and extramural invasion considered present). Those from Glasgow Royal also had elastica staining performed for venous invasion. The fifth edition of TNM staging system was used, consistent with the Royal College of Pathologists reporting guidelines during the time period studied. Clinical risk was assessed using the Petersen index to indicate low- and high-risk TNM stage II disease:^5^ a score of 1 was assigned to venous invasion or peritoneal involvement; a score of 2 was assigned to tumour perforation. Individuals with a Petersen index of ≥2 were considered high risk. Emergency surgery was defined as unplanned surgery within 5-days of index hospital admission. Modified Glasgow Prognostic score (mGPS) was calculated as previously described^21^ using serum C-reactive protein (CRP) and albumin levels measured in the 30 days preceding elective surgery, and on day of admission for emergency surgery. Data regarding adjuvant chemotherapy was not available for this cohort.

#### TransSCOT cohort

The TransSCOT cohort comprised 2912 patients from the SCOT study of adjuvant chemotherapy, with study criteria and clinicopathological characteristics previously described.^18^ Briefly, the cohort comprised of patients with stage III and high-risk stage II (one or more of T4 disease, tumour obstruction with or without perforation of the primary tumour preoperatively, fewer than ten lymph nodes harvested, poorly differentiated histology, perineural invasion or extramural venous or lymphatic invasion), treated with FOLFOX or CAPOX adjuvant chemotherapy randomised to 3- or 6-months’ duration. Tumours were staged using 7th edition of TNM. Date and site of recurrence and cause of death were crosschecked using electronic case records for both cohorts.

### Assessment of the tumour microenvironment

Whole H&E-stained sections of the deepest point of invasion were used for scoring the tumour microenvironment. Slides were scanned onto Slidepath Digital Image Hub, version 4.0.1 (Leica Biosystems, UK) using a Hamamatsu NanoZoomer at x20 magnification (Welwyn Garden City, UK). GMS combines KM and TSP assessment, as described previously.^11^ In brief, KM was scored semi-quantitatively at the invasive margin of the tumour as weak (none or only mild increase in inflammatory infiltrate) or strong (prominent inflammatory band or cup-like infiltrate). TSP was scored by assigning a percentage of the proportion of tumour-associated stroma present, including areas of mucin, at ×20 magnification. This was then dichotomised to low (≤50% stroma) or high (>50% stroma). KM and TSP were then combined: strong KM, regardless of TSP, scored GMS 0; weak KM with low TSP scored GMS 1; and weak KM with high TSP scored GMS 2. TSP and KM were already available for a subset of 1343 patients in the TransSCOT cohort, as these were utilised for assessing the Phenotypic Subtypes. For all microenvironment scoring, 10% of cases were co-scored in a blinded manner with an intra-class correlation co-efficient of >0.7.

Immunohistochemistry for generic T-cell (CD3) and cytotoxic (CD8) T-cell densities within the invasive margin, tumour stroma and cancer cell nests had previously been performed and reported for a subset of the validation cohort.^9^ In addition, a composite CD3/CD8 score comprising respective densities in the tumour centre and invasive margin was calculated, ranging from 0 (both CD3 and CD8 low in both regions) to 4 (both high in both regions).

### Mutational analysis

Mutational analysis was performed on a subset of patients from the validation cohort (*n* = 251). DNA was extracted from FFPE sections by NHS Tayside diagnostics and stored at −80 °C. DNA concentration was determined using Qubit assays (Thermo Fisher Scientific, MA, USA) and samples with ≥150 ng DNA were included in the study. DNA was diluted to 4 ng/µl and transferred to barcoded library tubes. Sequencing was performed by the Glasgow Precision Oncology Laboratory (GPOL) using the GPOL 151 CORE Cancer gene panel and run on a HiSeq4000 (Illumina, CA, USA). Data for KRAS and BRAF were converted to mutation annotation format and analysed using BiocManager maftools package in RStudio (R Studio, Inc, MA, USA).

### Statistical analysis

All data were subsequently analysed using SPSS version 25.0 (IBM SPSS). Kaplan–Meier and log-rank analysis compared survival adjusted for T-stage, N-stage and treatment duration, where appropriate. Hazard ratios (HR) and confidence intervals (CI) were calculated from univariate Cox regression survival analysis. Multivariable survival analysis using a backward conditional elimination model and a statistical significance threshold of *p* value < 0.1 was performed to identify independent prognostic biomarkers. Text results are reported as HR, 95% CI for GMS 0 vs GMS 2, but *p* value given is for log-rank analysis of overall trend. Pearson Chi-squared test was used to test associations between categorical variables and GMS. A Cox proportional hazard (PH) interaction model was performed to assess interactions between GMS and treatment type/duration. The study conformed to the REMARK guidelines^22^ and statistical significance was set at *p* value < 0.05.

## Results

### Validation cohort

In the validation cohort, there were 862 patients with TNM I–III CRC. Clinicopathological characteristics are presented in Table 1. Sixty percent of patients were younger than 75 years at time of surgery, and 35% were node positive. Fifty-eight percent had low-risk disease, while 42% had high-risk disease. Of the high-risk group, 61 were high-risk TNM II, whereas 302 were TNM III. Three hundred (35%) patients were GMS 0, 424 (49%) patients GMS 1 and 138 (16%) patients GMS 2. Median follow-up for all patients was 7.96 years (range: 2.3–11.1). There were 554 deaths and 271 patients developed recurrence.

**Table 1:** Relapse-free survival in stage I–III colorectal cancer and associations of clinicopathological features with GMS in patients from the validation cohort (*n* = 862).

Clinicopathological characteristics		Disease-free survival				Relapse-free survival				GMS category						
	*N* (%)^a^	Univariate HR (95% CI)	*P*	Multivariate HR (95% CI)	*P*	Univariate HR (95% CI)	*P*	Multivariate HR (95% CI)	*P*	0 (*n* = 300) *N* (%)^a^		1 (*n* = 424) *N* (%)		2 (*n* = 138) *N* (%)		*Pearson X*^*2*^
Age																
≤64	245 (28)									80	(27)	111	(26)	54	(39)	**0.04**
65–74	276 (32)									98	(33)	138	(33)	40	(29)	
≥75	341 (40)	1.66 (1.48–1.86)	**<0.001**	1.71 (1.51–1.95)	**<0.001**	1.10 (0.95–1.28)	0.20	–	–	122	(41)	175	(41)	44	(32)	
Gender																
Female	419 (49)									151	(50)	207	(49)	61	(44)	0.27
Male	443 (51)	1.12 (0.95–1.33)	0.18	–	–	1.15 (0.91–1.47)	0.24	–	–	149	(50)	217	(51)	77	(56)	
Presentation																
Elective	686 (80)									260	(87)	319	(75)	107	(78)	**0.002**
Emergency	175 (20)	1.55 (1.27–1.89)	**<0.001**	–	0.17	1.94 (1.49–2.53)	**<0.001**	–	0.10	39	(13)	105	(25)	31	(22)	
TNM																
I–II (low risk)	499 (58)									201	(67)	244	(58)	54	(39)	**<0.001**
II–III (high risk)	363 (42)	1.58 (1.33–1.87)	**<0.001**^b^	–	–	3.02 (2.36–3.87)	**<0.001**^b^	–	–	99	(33)	180	(42)	84	(61)	
T-stage																
T1	42 (5)									28	(9)	13	(3)	1	(1)	**<0.001**
T2	113 (13)									65	(22)	39	(9)	9	(7)	
T3	488 (57)									150	(50)	260	(61)	78	(56)	
T4a	179 (20)									43	(14)	93	(22)	43	(31)	
T4b	40 (5)	1.30 (1.15–1.46)	**<0.001**	1.25 (1.08–1.44)	**0.003**	1.81 (1.51–2.16)	**<0.001**	1.46 (1.17–1.82)	**0.001**	14	(5)	19	(5)	7	(6)	
N-stage																
N0	556 (65)									217	(73)	272	(64)	67	(49)	**<0.001**
N1	218 (25)									66	(22)	105	(25)	47	(34)	
N2	84 (10)	1.38 (1.22–1.55)	**<0.001**	1.31 (1.14–1.51)	<**0.001**	1.96 (1.68–2.30)	**<0.001**	1.58 (1.32–1.90)	<**0.001**	16	(5)	45	(11)	23	(17)	
Site																
Colon	650 (75)									206	(69)	345	(81)	99	(72)	0.08
Rectum	212 (25)	0.93 (0.76–1.13)	0.44	–	–	0.96 (0.73–1.27)	0.78			94	(31)	79	(19)	39	(28)	
Differentiation																
Well/mod	775 (90)									271	(90)	379	(89)	125	(91)	0.95
Poor	87 (10)	1.36 (1.04–1.79)	**0.03**	–	0.46	1.61 (1.13–2.30)	**0.01**	–	0.87	29	(10)	45	(11)	13	(9)	
Venous invasion																
Absent	589 (68)									226	(75)	290	(69)	73	(53)	**<0.001**
Present	273 (32)	1.38 (1.15–1.65)	**<0.001**	–	0.18	1.86 (1.46–2.37)	**<0.001**	1.34 (1.01–1.76)	**0.04**	74	(25)	134	(31)	65	(47)	
Tumour budding																
Absent	618 (72)									219	(73)	304	(72)	95	(69)	0.39
Present	244 (28)	1.04 (0.86–1.25)	0.70	–	–	1.33 (1.03–1.71)	**0.03**	–	0.15	81	(27)	120	(28)	43	(31)	
KRAS status (*n* = 212)																
Wild-type	111 (52)									36	(55)	53	(50)	22	(55)	0.86
Mutant	101 (48)	1.16 (0.84–1.59)	0.37	–	–	1.08 (0.72–1.61)	0.71	–	–	29	(45)	54	(50)	18	(45)	
BRAF status (*n* = 212)																
Wild-type	182 (86)									54	(83)	91	(85)	37	(93)	0.21
Mutant	30 (14)	1.03 (0.66–1.59)	0.90	–	–	0.91 (0.51–1.64)	0.76	–	–	11	(17)	16	(15)	3	(7)	
MMR																
Proficient	686 (82)									230	(78)	342	(83)	114	(84)	0.10
Deficient	155 (18)	0.99 (0.80–1.23)	0.94	–	–	0.80 (0.57–1.11)	0.18	–	–	64	(22)	69	(17)	22	(16)	
mGPS																
0	386 (55)									152	(59)	175	(52)	59	(54)	0.19
1	201 (29)									68	(27)	102	(30)	31	(28)	
2	115 (16)	1.59 (1.40–1.79)	**<0.001**	1.52 (1.34–1.73)	**<0.001**	1.69 (1.43–1.99)	**<0.001**	1.59 (1.34–1.90)	**<0.001**	37	(14)	59	(18)	19	(17)	
GMS																
0	300 (35)									–		–		–		–
1	424 (49)											–		–		
2	138 (16)	1.24 (1.09–1.40)	**0.001**	1.24 (1.07–1.43)	**0.004**	1.76 (1.48–2.08)	<**0.001**	1.53 (1.26–1.86)	**<0.001**	–		–		–		

^a^Percentages rounded to nearest whole number and may not total 100%

^b^Not included in multivariate model as T-stage and N-stage included separately.

Emboldened values indicates *p* value < 0.05.

**Fig. 1:**
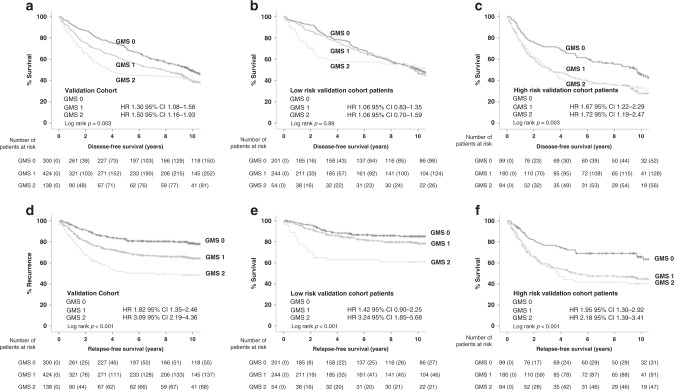
GMS can stratify recurrence and survival according to disease risk in the validation cohort. **a**–**c** Kaplan–Meier curve showing associations between GMS and DFS in the **a** full cohort (*n* = 862), **b** “low-risk” colorectal cancer (*n* = 499) and **c** “high-risk” colorectal cancer (*n* = 363). **d**–**e** Kaplan–Meier curve showing associations between GMS and RFS in the **a** full cohort (*n* = 862), **b** “low-risk” colorectal cancer (*n* = 499) and **c** “high-risk” colorectal cancer (*n* = 363).

Associations between GMS and DFS were assessed (Table 1). GMS stratified survival in the whole cohort for DFS with 5-year DFS for GMS 0, 1 and 2 of 71%, 58 and 46%, respectively, (GMS 0 v GMS 2: HR 1.50 95% CI 1.16–1.93, *p* = 0.002; Fig. 1a). On multivariate analysis for DFS, GMS remained independent (*p* = 0.004) of age (*p* < 0.001), T-stage (*p* = 0.003), N-stage (*p* < 0.001) and mGPS (*p* < 0.001). Subgroup analysis was performed according to clinical risk (low risk: TNM I–II and Petersen Index <2; high risk: TNM II and Petersen index ≥2 or TNM III) and primary tumour site (Table 2). While GMS did not stratify survival in low-risk disease (Fig. 1b), high-risk disease was stratified with 5-year DFS for GMS 0, 1 and 2 of 66%, 43 and 38%, respectively, (GMS 0 v GMS 2: HR 1.72 95% CI 1.19–2.47, *p* = 0.003; Fig. 1c). In addition, GMS was able to stratify 5-year DFS for colon cancer with GMS 0, 1 and 2 of 72%, 58 and 45%, respectively, (GMS 0 v GMS 2: HR 1.57 95% CI 1.16–2.12, *p* = 0.004; Fig. S1A), but not rectal cancer (S1B).

**Table 2:** Survival for GMS according to low- and high-risk disease and location of cancer in the validation cohort (*n* = 862).

GroupGMS category		Disease-free survival				Relapse-free survival			
	*N*	5-year DFS (%; SE)	Events (*n* = 541)	HR (95% CI)	*P*	5-year RFS (%; SE)	Events (*n* = 271)	HR (95% CI)	*P*
Full cohort				*Trend*	*0.003*			*Trend*	*<0.001*
0	300	71 (3)	168	1.0 (reference)		83 (2)	61	1.0 (reference)	
1	424	58 (2)	281	1.30 (1.08–1.58)	**0.007**	70 (2)	141	1.82 (1.35-2.46)	**<0.001**
2	138	46 (4)	92	1.50 (1.16–1.93)	**0.002**	51 (4)	69	3.09 (2.19-4.36)	**<0.001**
TNM I–II (low risk)				*Trend*	*0.89*			*Trend*	*<0.001*
0	201	73 (3)	113	1.0 (reference)		88 (2)	29	1.0 (reference)	
1	244	70 (3)	148	1.06 (0.83–1.35)	0.65	84 (2)	49	1.42 (0.90–2.25)	0.13
2	54	57 (7)	29	1.06 (0.70–1.59)	0.79	63 (7)	21	3.24 (1.85–5.68)	**<0.001**
TNM II–III (high risk)				*Trend*	*0.003*			*Trend*	*0.001*
0	99	66 (5)	55	1.0 (reference)		72 (5)	32	1.0 (reference)	
1	180	43 (4)	133	1.67 (1.22–2.29)	**0.001**	51 (4)	92	1.95 (1.30–2.92)	**0.001**
2	84	38 (5)	63	1.72 (1.19–2.47)	**0.003**	43 (6)	48	2.18 (1.39–3.41)	**0.001**
Colon cancer				*Trend*	*0.004*			*Trend*	*<0.001*
0	206	72 (3)	113	1.0 (reference)		84 (3)	41	1.0 (reference)	
1	345	58 (3)	233	1.38 (1.10–1.73)	**0.005**	69 (3)	115	1.88 (1.32–2.68)	**0.001**
2	99	45 (5)	67	1.57 (1.16–2.12)	**0.004**	51 (5)	49	3.15 (2.08–4.77)	**<0.001**
Rectal cancer				*Trend*	*0.46*			*Trend*	*0.003*
0	94	68 (5)	55	1.0 (reference)		80 (4)	20	1.0 (reference)	
1	79	62 (5)	48	1.07 (0.73–1.58)	0.72	72 (5)	26	1.66 (0.92–2.97)	0.09
2	39	46 (8)	25	1.35 (0.84–2.17)	0.21	51 (8)	20	2.95 (1.58–5.48)	**0.001**

*DFS* disease-free survival, *RFS* relapse-free survival.

Emboldened values indicates *p* value < 0.05

Next, associations between GMS and RFS were assessed (Table 1). GMS significantly stratified RFS for the whole cohort with 5-year RFS of 83%, 70 and 51% for GMS 0, 1 and 2, respectively, (GMS 0 v GMS 2: HR 3.09 95% CI 2.19–4.36, *p* < 0.001, Fig. 1d). On multivariate analysis for RFS, GMS remained associated with survival (*p* < 0.001) independent of T-stage (*p* = 0.001), N-stage (*p* < 0.001), venous invasion (*p* = 0.04) and mGPS (*p* < 0.001). In low-risk disease (Table 2), 5-year RFS was 88%, 84 and 63% for GMS 0, 1 and 2, respectively, with GMS 2 associated with significantly worse RFS (GMS 0 v GMS 2: HR 3.24 95% CI 1.85–5.68, *p* < 0.001, Fig. 1e). In high-risk disease (Table 2), 5-year RFS was 72%, 51 and 43% for GMS 0, 1 and 2, respectively, and GMS 0 had significantly better RFS (GMS 0 v GMS 2: HR 2.18 95% CI 1.39–3.41, *p* = 0.001, Fig. 1f). On subgroup analysis by disease site (Table 2), GMS stratified RFS in patients with colon cancer (*n* = 650), with 5-year RFS for GMS 0, 1 and 2 of 84%, 69 and 51%, respectively, (GMS 0 v GMS 2: HR 3.15 95% CI 2.08–4.77, *p* < 0.001, Fig. S1C), and rectal cancer (*n* = 212), with 5-year RFS for GMS 0, 1 and 2 of 80%, 72 and 51%, respectively, (GMS 0 v GMS 2: HR 2.95 95% CI 1.58–5.48, *p* = 0.001 Fig. S1D).

Overall (OS) and cancer-specific survival (CSS) data were available for the validation cohort and these are displayed in Supplementary Tables S1–2 and Fig. S2. GMS was independently significant on multivariate analysis for OS (*p* < 0.01) and for CSS (*p* < 0.001). On subgroup analysis for OS, the results were comparable to DFS, with GMS stratifying OS for the full cohort (GMS 0 v GMS 2: HR 1.50 95% CI 1.17–1.93, *p* = 0.003), high-risk disease (GMS 0 v GMS 2: HR 1.67 95% CI 1.18–2.38, *p* = 0.009), and colon cancer (GMS 0 v GMS 2: HR 1.49 95% CI 1.11–2.00, *p* = 0.02), but not low-risk disease or rectal cancer. Likewise, the subgroup analysis for CSS was similar to that for RFS, with GMS stratifying CSS for the full cohort (GMS 0 v GMS 2: HR 3.55 95% CI 2.44–5.16, *p* < 0.001), low-risk disease (GMS 0 v GMS 2: HR 3.94 95% CI 2.10–7.39, *p* < 0.001), high-risk disease (GMS 0 v GMS 2: HR 2.34 95% CI 1.46–3.76, *p* = 0.001), colon cancer (GMS 0 v GMS 2: HR 3.36 95% CI 2.14–5.27, *p* < 0.001) and rectal cancer (GMS 0 v GMS 2: HR 4.07 95% CI 2.08–7.96, *p* < 0.001).

The relationship between GMS and pattern of recurrence was examined (Supplementary Table S3). GMS 1 and 2 were associated with higher risk of recurrence (GMS 015%, GMS 126%, GMS 241%, *p* < 0.001.) Although this was predominantly due to an increase in risk of distant recurrence, patients with GMS 2 were more likely to develop local recurrence compared to GMS 0 or 1.

Furthermore, associations between GMS and CD3, CD8 and composite CD3/CD8 score were assessed (Table S4, *n* = 208). GMS was associated with individual T-cell densities in all locations and composite score, with highest density observed in GMS 0 and lowest density generally observed in GMS 2. Univariate survival analysis found comparable hazard ratios and confidence intervals for all immune cell markers. These were not combined in multivariate analysis as all included analysis of an inflammatory variable and would therefore be mutually exclusive.

The relationship between GMS and clinicopathological characteristics was examined (Table 1). Increasing GMS was significantly associated with younger age (*p* = 0.04), emergency presentation (*p* = 0.002), high-risk TNM (*p* < 0.001), higher T- and N-stage (both *p* < 0.001), peritoneal involvement (*p* < 0.001) and venous invasion (*p* < 0.001). There were no significant associations between GMS and KRAS or BRAF mutations. Neither were these mutations significant for survival in the validation cohort for those with results available for analysis.

### TransSCOT cohort

In the TransSCOT cohort, there were 2912 TNM II–III patients, all of whom received FOLFOX (*n* = 846) or CAPOX (*n* = 2066) adjuvant chemotherapy for at least 3 months. 383 (13%) patients were GMS 0, 1866 (64%) patients GMS 1, and 663 (23%) patients GMS 2. Median follow-up was 3.0 years (range: 0.0–7.0) with 755 DFS events. Cohort characteristics shown in Table 3 were similar to those in the full SCOT trial and therefore representative of this population.^18^

**Table 3:** Disease-free survival in the TransSCOT cohort and associations of clinicopathological features with GMS (*n* = 2912).

Clinicopathological characteristics		Disease-free survival				GMS category						
	*N* (%)^a^	Univariate HR (95% CI)	*P*	Multivariate HR (95% CI)	*P*	0 (*n* = 383) *N* (%)^a^		1 (*n* = 1867) *N* (%)		2 (*n* = 663) *N* (%)		*Pearson X*^*2*^
Gender												
Female	1135 (39)					156	(41)	716	(38)	263	(40)	0.63
Male	1778 (61)	0.96 (0.83–1.11)	0.60	–	–	227	(59)	1151	(62)	400	(60)	
T-stage												
T1	78 (3)					19	(5)	55	(3)	4	(1)	**<0.001**
T2	250 (9)					59	(15)	160	(9)	31	(5)	
T3	1696 (58)					227	(60)	1131	(61)	338	(51)	
T4	889 (30)	1.70 (1.51–1.91)	**<0.001**	1.74 (1.53–1.98)	**<0.001**	78	(20)	521	(28)	290	(43)	
N-stage												
N0	556 (19)					79	(21)	362	(19)	115	(17)	**0.002**
N1	1663 (57)					224	(58)	1086	(58)	353	(53)	
N2	694 (24)	1.75 (1.57–1.96)	**<0.001**	1.73 (1.48–2.03)	<**0.001**	80	(21)	419	(22)	195	(29)	
Site												
Colon	2402 (82)					310	(81)	1522	(81)	570	(86)	**0.021**
Rectum	511 (18)	0.69 (0.56–0.85)	**<0.001**	–	0.12	73	(19)	345	(19)	93	(14)	
Risk group												
T1-3/N1 (lower risk)	1284 (55)					202	(66)	861	(57)	221	(40)	**<0.001**
T4 and/or N2 (higher risk)	1073 (45)	2.45 (2.08–2.88)	**<0.001**	–	0.13	102	(34)	644	(43)	327	(60)	
Adjuvant therapy												
FOLFOX	846 (29)					120	(31)	526	(28)	200	(30)	0.36
CAPOX	2067 (71)	1.08 (0.92–1.27)	0.32	–	–	263	(69)	1341	(72)	463	(70)	
Treatment time												
3 months	1469 (50)					194	(51)	956	(51)	319	(48)	0.39
6 months	1444 (50)	1.01 (0.88–1.16)	0.85	–	–	180	(49)	911	(49)	344	(52)	
GMS												
0	300 (35)					–		–		–		–
1	424 (49)					–		–		–		
2	138 (16)	1.48 (1.32–1.68)	**<0.001**	1.28 (1.12–1.47)	**<0.001**	–		–		–		

^a^Percentages rounded to nearest whole number and may not total 100%.

Emboldened values indicates *p* value < 0.05

**Fig. 2:**
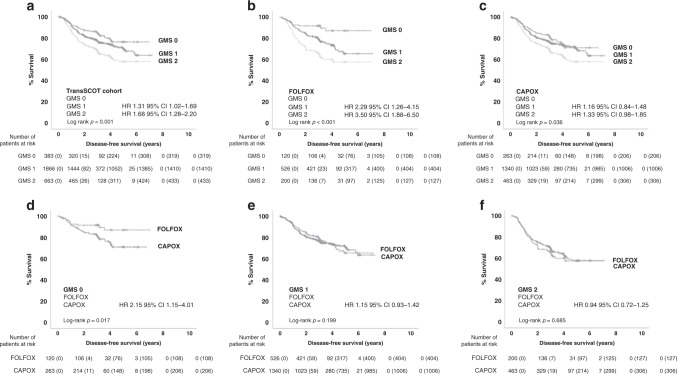
GMS can identify patient response to adjuvant chemotherapy within the TransSCOT cohort. **a** Kaplan–Meier curve showing associations between GMS and DFS in the full cohort (*n* = 2912). **b**, **c** Kaplan–Meier curves showing associations between GMS and DFS in patients receiving **b** FOLFOX (*n* = 846) or **c** CAPOX (*n* = 2066) adjuvant chemotherapy. **d**–**f** Kaplan–Meier curves showing associations between chemotherapy type and DFS in patients with **d** GMS 0 (*n* = 383), **e** GMS 1 (*n* = 1866) or **f** GMS 2 (*n* = 663).

In the full cohort, GMS significantly stratified survival with a 5-year DFS for GMS 0, 1 and 2 of 69%, 63 and 53%, respectively, (GMS 0 v GMS 2: HR 1.68 95% CI 1.28–2.20, *p* < 0.001, Fig. 2a). Patients were then stratified for disease site. In patients with colon cancer (*n* = 2402), GMS stratified survival, with 5-year DFS for GMS 0, 1 and 2 of 76%, 66 and 56%, respectively, (GMS 0 v GMS 2: HR 2.20 95% CI 1.64–2.94, *p* < 0.001, Fig. S3A). For patients with rectal cancer (*n* = 510), GMS did not associate with DFS (GMS 0 v GMS 2: HR 1.74 95% CI 0.85–3.57, *p* = 0.130, Fig. S3B). On multivariate analysis (Table 3), T-stage (*p* < 0.001), N-stage (*p* < 0.001) and GMS (*p* < 0.001) independently associated with DFS. Furthermore, GMS associated with higher T-stage (*p* < 0.001), higher N-stage (*p* = 0.002), colonic site (*p* = 0.021) and higher-risk TNM III disease (*p* < 0.001).

The interaction between GMS and adjuvant chemotherapy type and duration was investigated (Table S5). Multivariate Cox PH analysis was performed, demonstrating a significant interaction between GMS and chemotherapy type (*p* = 0.01) but not duration (*p* = 0.64). As an interaction was seen between GMS and chemotherapy type, associations with DFS where stratified for FOLFOX and CAPOX. For patients receiving FOLFOX, the association with DFS was strengthened with a 5-year DFS for GMS 0, 1 and 2 of 88%, 62 and 54%, respectively, (GMS 0 v GMS 2: HR 3.50 95% CI 1.88–6.50, *p* < 0.001, Fig. 2B). However, for patients receiving CAPOX these associations were dampened with a 5-year DFS for GMS 0, 1 and 2 of 62%, 63 and 53%, respectively, (GMS 0 v GMS 2: HR 1.33 95% CI 0.98–1.85, *p* = 0.07, Fig. 2c). As associations with DFS were strengthened in the FOLFOX-treated patients, patients were stratified by GMS category to assess if any group responded more favourably to one particular therapy. Patients with GMS 0 significantly benefited from FOLFOX over CAPOX, with 5-year DFS of 88% v 62% (HR 2.23 95% CI 1.19–4.16, *p* < 0.001, Fig. 2d). However, no difference in DFS was seen for GMS 1 with 5-year DFS for FOLFOX and CAPOX of 62% v 63% (HR 1.08 95% CI 0.88–1.33, *p* = 0.21, Fig. 2e) or GMS 2 with 5-year of 54% v 53%, respectively, (HR 0.90 95% CI 0.68–1.19, *p* = 0.68, Fig. 2f). To ensure that the interaction between GMS 0 and chemotherapy type was not inadvertently due to one group receiving a longer course of chemotherapy than another, a further test of association was performed between type and duration of chemotherapy in the GMS 0 subgroup. There was no significant association between chemotherapy type and duration in this subgroup (*p* = 0.11; Table S6).

**Fig. 3:**
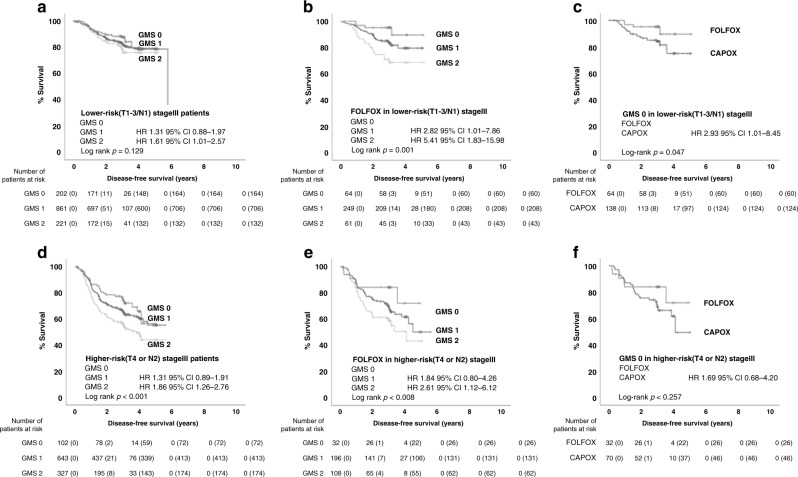
GMS, prognosis and response to adjuvant chemotherapy in lower- and higher-risk stage III patients from the TransSCOT cohort (*n* = 2356). **a**, **d** Kaplan–Meier curves showing associations between GMS and DFS in **a** lower-risk (*n* = 1284) and **b** higher-risk stage III patients (*n* = 1072). **b**, **e** Kaplan–Meier curves showing associations between GMS and DFS in **b** lower-risk (*n* = 374) and **e** higher-risk (*n* = 336) stage III patients receiving FOLFOX adjuvant chemotherapy. **c**, **f** Kaplan–Meier curves showing associations between chemotherapy type and DFS in GMS 0 patients within the **c** lower-risk (*n* = 202) and **f** higher-risk (*n* = 102) stage III groups.

To assess the utility of GMS in lower- and higher-risk TNM III disease, as defined by the SCOT trial, TNM III patients were stratified into lower risk (T1-3/N1) and higher-risk (T4 or N2) groups. GMS did not stratify DFS in the lower-risk patients (GMS 0 v GMS 2: HR 1.61 95% CI 1.01–2.57, *p* = 0.13, Fig. 3a), but significantly stratified higher-risk patients (GMS 0 v GMS 2: HR 1.86 95% CI 1.26–2.76, *p* = 0.002, Fig. 3b). Next, interactions with type and duration of chemotherapy were assessed (Table S5). GMS did not interact with duration in either group. GMS interacted with type of chemotherapy in lower-risk patients (*p* = 0.005) but not higher-risk patients (*p* = 0.61). For patients receiving FOLFOX, GMS stratified DFS in both the lower risk (GMS 0 v GMS 2: HR 5.41 95% CI 1.83–15.98, *p* = 0.001, Fig. 3c) and higher-risk disease (GMS 0 v GMS 2: HR 2.61 95% CI 1.12–6.12, *p* = 0.03, Fig. 3d). However, when assessing chemotherapy type within TNM III patients with GMS 0, patients benefited from FOLFOX over CAPOX chemotherapy in lower risk (HR 2.94 95% CI 1.02–8.47, *p* = 0.04, Fig. 3e), but not higher-risk disease (HR 1.82 95% CI 0.75–4.47, *p* = 0.18, Fig. 3f).

## Discussion

The results presented from both the expanded validation and TransSCOT cohorts validate the utility of GMS as an independent prognostic marker in colorectal cancer. This represents the largest study to date investigating a combination scoring system of peritumoural inflammation and mesenchymal phenotype. Other microenvironment scores have been proposed, such as: the Immunoscore,^10^ which uses immunohistochemical staining for CD3 and CD8 and a digital pathology software platform to evaluate immune infiltrates; colorectal cancer intrinsic subtypes (CRIS), which uses genetic testing of a number of genes implicated in colorectal cancer to stratify tumour behaviour/response;^23^ the Phenotypic Subtypes, which have already been addressed in this paper, combining KM, TSP and Ki67 immunohistochemistry; and the image-based consensus molecular subtype, which uses artificial intelligence analysis of digital pathology slides.^24^ GMS has advantages over these scores in that it does not require the use of additional immunohistochemical staining, genetic testing or digital pathology, as it can be performed on the H&E slides that are used in routine clinical practice for TNM staging. Furthermore, in the subset of patients with both GMS and IHC available, GMS was strongly associated with CD3 and CD8. In addition, there were similar univariate RFS for all inflammatory scores. This again supports the GMS as a clinically applicable prognostic score in patients with colorectal cancer.

In the validation cohort, GMS stratified survival of both low-risk and high-risk patients, in terms of stage and Petersen index, with GMS 2 highlighting a group of low-risk patients that may benefit from additional adjuvant therapy. GMS 2 may therefore be considered for addition to the current list of high-risk pathological features discussed at multidisciplinary team meetings to guide ongoing management. GMS 1 defined a group of patients with neither high immunity, nor high TSP who have an intermediate outcome that varies with disease stage, with better survival in low-risk disease, but worse survival in high-risk disease. Whereas, GMS 0 indicated a group of patients that had a good clinical outcome regardless of disease stage, in keeping with previous research in high immune tumours.^25^

Patients with GMS 2 appear to reflect a particularly poor prognostic group, with a clear reduction in not only OS, but also DFS, CSS and RFS. Previous work has proposed that such a phenotype, characterised by high stromal infiltration and weak immune response, reflects a mesenchymal subtype with poor prognosis and increased risk of recurrence.^20^ In the present study, patients with GMS 2 had the highest risk of both local and distant recurrence. Kaplan–Meier curves showed an early and sustained drop in survival of patients with GMS 2, particularly over the first two years of follow-up, reflecting the time period in which patients are most likely to develop recurrent metastatic disease.^26^ In contrast, survival continued to decline gradually throughout follow-up in patients with GMS 0 and 1, likely reflecting alternative causes of death in these groups. Indeed, whether patients with GMS 2 may benefit from enhanced surveillance strategies would be of interest.

The association of GMS with chemotherapy regimen was explored in the TransSCOT cohort. GMS survival stratification in the TransSCOT cohort was similar to that in the validation cohort. GMS 2 patients derived less benefit from adjuvant chemotherapy independent of regimen used or risk stratification. GMS 1 patients did not respond better to any particular chemotherapy type but had an intermediate survival outcome. However, for GMS 0 patients receiving FOLFOX, survival was significantly better than those receiving CAPOX, especially in lower-risk TNM III. This did not appear to reflect differences in duration of chemotherapy.

Whilst further validation is required, the results suggest that those with higher peritumoural inflammation have different clinical outcomes depending on which form of 5-FU-based chemotherapy is administered. FOLFOX was shown to offer a more favourable outcome in the presence of high peritumoural inflammation (GMS 0). However, in the absence of such an infiltrate (both GMS 1 and GMS 2), there was no survival difference.

Previous studies have reported that colorectal cancer patients receiving chemotherapy have better outcomes if they have higher tumour-infiltrating lymphocytes.^25,27,28^ However, there are no previously published studies that have compared the efficacy of FOLFOX vs CAPOX depending on peritumoural inflammation. The link between high KM and type of chemotherapy was demonstrated by our group when investigating the 1343 TransSCOT patients studied for the Phenotypic Subtypes study.^20^ Since the assessment of Ki67 did not add to this differentiation, only the GMS was performed on the full TransSCOT cohort. There is, therefore, paucity of data as to the mechanism underlying this effect and further investigation is required. One hypothesis is that the high levels of immune cells hamper the final stage of capecitabine metabolism, inhibiting its cytotoxic effect and therefore dampening the effect of CAPOX. However, as previously stated, patients with higher peritumoural inflammation have better outcomes on adjuvant chemotherapy and so this explanation holds little weight. Alternatively, the administration of intravenous 5-FU in the FOLFOX regimen may result in better bioavailability of the active metabolite, fluoro-deoxyuridine monophosphate, than oral Capecitabine and this effect would be more pronounced in the higher immune group. Further still, Folinic Acid (Leucovorin) is administered as part of the FOLFOX regimen as it has been found to enhance the antitumour effects of 5-FU.^29^ Folinic acid is an intravenous folate and is also used to supplement vitamin B9, which can protect against bone marrow suppression^30^ and this may protect FOLFOX patients with high peritumoural inflammation against the immunosuppressive side effects of chemotherapy. However, there are no studies to date exploring this phenomenon.

Pagès et al.^31^ recently published results comparing the Immunoscore in the French cohort of the IDEA study, finding that those with higher antitumour immunity might benefit from longer course mFOLFOX6. While the results of the TransSCOT cohort validate the use of FOLFOX over CAPOX in this patient group, there was no association between duration of treatment and GMS status.

GMS was unable to significantly stratify disease-free survival of patients with rectal cancer in either cohort. There were smaller numbers in this subgroup, and this may be one reason for the lack of stratification. In addition, a proportion of patients may have received neoadjuvant radiotherapy, which would impact upon post-operative tumour microenvironment assessment. However, there were significant differences in survival between GMS 0 vs GMS 2 for both RFS and CSS in the validation cohort; this requires further study in additional patient cohorts.

Lack of mutational data represents a limitation of this study. This could be strengthened by combining the GMS with mutational analysis and this represents one of the future directions of this group. A further limitation of the current study is the lack of overall and cancer-specific survival data in the TransSCOT cohort. However, as shown in the validation cohort, the curves were very similar for DFS and overall survival and therefore, DFS can be considered a reasonable primary endpoint.

In conclusion, the present study validates the prognostic utility of the Glasgow Microenvironment Score. The poor outcome in low-risk disease of GMS 2 indicates that this subgroup may not derive benefit from current therapies. However, GMS 2 may be considered an additional high-risk feature that warrants consideration for novel therapies. Conversely, GMS 0 in high-risk patients highlights a subgroup that may benefit most from current therapies. This survival effect was strengthened in patients receiving FOLFOX but dampened in patients receiving CAPOX. Therefore, GMS could be a useful tool to aid both prognostic and therapeutic decision making in clinical practice alongside TNM staging. GMS should be further assessed in the context of prospective randomised clinical trials.

## Supplementary information

Supplementary data

## Data Availability

The datasets that formed the basis of this article are contained in the University of Glasgow’s MVLS institute and are continually being updated with ongoing research. They contain patient sensitive information and therefore cannot be made available on a public repository.
