# Supplementary material for: The Glasgow Microenvironment Score associates with prognosis and adjuvant chemotherapy response in colorectal cancer
Source: Br J Cancer. 2020 Nov 23;124(4):786–96. doi: 10.1038/s41416-020-01168-x (PMC7884404; doi:10.1038/s41416-020-01168-x)

**Supplementary Table S1. Overall and cancer-specific survival in stage I-III colorectal cancer in patients from the validation cohort (*N*=862).**

| Clinicopathological characteristics | | Overall survival | | | | Cancer-specific survival | | | |
| --- | --- | --- | --- | --- | --- | --- | --- | --- | --- |
|  | *N* (%)^a^ | Univariate HR (95% CI) | *P* | Multivariate HR (95% CI) | *P* | Univariate HR (95% CI) | *P* | Multivariate HR (95% CI) | *P* |
| Age |  |  |  |  |  |  |  |  |  |
| ≤64 | 245 (28) |  |  |  |  |  |  |  |  |
| 65-74 | 276 (32) |  |  |  |  |  |  |  |  |
| ≥75 | 341 (40) | 1.78 (1.59-2.00) | **<0.001** | 1.88 (1.65-2.13) | **<0.001** | 1.11 (0.95-1.30) | 0.20 | – | – |
| Gender |  |  |  |  |  |  |  |  |  |
| Female | 419 (49) |  |  |  |  |  |  |  |  |
| Male | 443 (51) | 1.18 (0.99-1.39) | 0.06 | 1.30 (1.07-1.58) | **0.007** | 1.23 (0.95-1.60) | 0.11 | – | – |
| Presentation |  |  |  |  |  |  |  |  |  |
| Elective | 686 (80) |  |  |  |  |  |  |  |  |
| Emergency | 175 (20) | 1.48 (1.21-1.82) | **<0.001** | – | 0.23 | 1.87 (1.40-2.49) | **<0.001** | – | 0.28 |
| TNM |  |  |  |  |  |  |  |  |  |
| I-II (low risk) | 499 (58) |  |  |  |  |  |  |  |  |
| II-III (high risk) | 363 (42) | 1.49 (1.26-1.76) | **<0.001**^b^ | – | – | 3.22 (2.46-4.22) | **<0.001**^b^ | – | – |
| T-stage |  |  |  |  |  |  |  |  |  |
| T1 | 42 (5) |  |  |  |  |  |  |  |  |
| T2 | 113 (13) |  |  |  |  |  |  |  |  |
| T3 | 488 (57) |  |  |  |  |  |  |  |  |
| T4a | 179 (20) |  |  |  |  |  |  |  |  |
| T4b | 40 (5) | 1.23 (1.10-1.38) | **<0.001** | 1.19 (1.03-1.36) | **0.02** | 1.85 (1.52-2.25) | **<0.001** | 1.43 (1.13-1.81) | **0.002** |
| N-stage |  |  |  |  |  |  |  |  |  |
| N0 | 556 (65) |  |  |  |  |  |  |  |  |
| N1 | 218 (25) |  |  |  |  |  |  |  |  |
| N2 | 84 (10) | 1.31 (1.17-1.48) | **<0.001** | 1.29 (1.12-1.48) | <**0.001** | 1.99 (1.69-2.34) | **<0.001** | 1.55 (1.29-1.87) | <**0.001** |
| Site |  |  |  |  |  |  |  |  |  |
| Colon | 650 (75) |  |  |  |  |  |  |  |  |
| Rectum | 212 (25) | 0.91 (0.75-1.11) | 0.35 | – | – | 1.04 (0.78-1.39) | 0.80 |  |  |
| Differentiation |  |  |  |  |  |  |  |  |  |
| Well/mod | 775 (90) |  |  |  |  |  |  |  |  |
| Poor | 87 (10) | 1.32 (1.01-1.73) | 0.05 | – | 0.40 | 1.69 (1.16-2.47) | **0.01** | – | 0.63 |
| Venous invasion |  |  |  |  |  |  |  |  |  |
| Absent | 589 (68) |  |  |  |  |  |  |  |  |
| Present | 273 (32) | 1.38 (1.16-1.65) | **<0.001** | – | 0.18 | 2.06 (1.60-2.67) | **<0.001** | 1.48 (1.11-1.97) | **0.008** |
| Tumour budding |  |  |  |  |  |  |  |  |  |
| Absent | 618 (72) |  |  |  |  |  |  |  |  |
| Present | 244 (28) | 1.04 (0.87-1.25) | 0.67 | – | – | 1.33 (1.01-1.74) | **0.04** | – | 0.16 |
| KRAS status (*n*=212) |  |  |  |  |  |  |  |  |  |
| Wild-type | 111 (52) |  |  |  |  |  |  |  |  |
| Mutant | 101 (48) | 1.21 (0.87-1.67) | 0.26 | – | – | 1.11 (0.73-1.71) | 0.62 | – | – |
| BRAF status ( *n*=212) |  |  |  |  |  |  |  |  |  |
| Wild-type | 182 (86) |  |  |  |  |  |  |  |  |
| Mutant | 30 (14) | 0.85 (0.53-1.37) | 0.51 | – | – | 0.69 (0.35-1.38) | 0.30 | – | – |
| MMR |  |  |  |  |  |  |  |  |  |
| Proficient | 686 (82) |  |  |  |  |  |  |  |  |
| Deficient | 155 (18) | 1.03 (0.83-1.28) | 0.82 | – | – | 0.88 (0.62-1.25) | 0.46 | – | – |
| mGPS |  |  |  |  |  |  |  |  |  |
| 0 | 386 (55) |  |  |  |  |  |  |  |  |
| 1 | 201 (29) |  |  |  |  |  |  |  |  |
| 2 | 115 (16) | 1.58 (1.40-1.78) | **<0.001** | 1.51 (1.33-1.71) | **<0.001** | 1.75 (1.47-2.08) | **<0.001** | 1.61 (1.35-1.93) | **<0.001** |
|  |  |  |  |  |  |  |  |  |  |
| GMS  0  1  2 |  |  |  |  |  |  |  |  |  |
|  | 300 (35)  424 (49)  138 (16) |  |  |  |  |  |  |  |  |
|  |  | 1.23 (1.09-1.39) | **0.001** | 1.21 (1.05-1.39) | **0.009** | 1.88 (1.56-2.25) | <**0.001** | 1.63 (1.32-2.00) | **<0.001** |

^a^percentages rounded to nearest whole number and may not total 100% ^b^not included in multivariate model as T-stage and N-stage included separately

**Supplementary Table S2. Overall and Cancer-Specific Survival for GMS according to low- and high- risk disease and location of cancer in the validation cohort (*N*=862).**

| Group  GMS category |  | Overall Survival | | | | Cancer-specific Survival | | | |
| --- | --- | --- | --- | --- | --- | --- | --- | --- | --- |
|  | *N* | 5-year OS  (%; SE) | Events (*N*=554) | HR (95% CI) | *P* | 5-year CSS  (%; SE) | Events (*N*=235) | HR (95% CI) | *P* |
| Full cohort |  |  |  | *Trend* | *0.003* |  |  | *Trend* | *<0.001* |
| 0 | 300 | 69 (3) | 176 | 1.0 (reference) |  | 87 (2) | 48 | 1.0 (reference) |  |
| 1 | 424 | 58 (2) | 282 | 1.27 (1.05-1.53) | **0.015** | 72 (2) | 123 | 2.00 (1.43-2.79) | **<0.001** |
| 2 | 138 | 48 (4) | 96 | 1.50 (1.17-1.93) | **0.001** | 55 (4) | 64 | 3.55 (2.44-5.16) | **<0.001** |
|  |  |  |  |  |  |  |  |  |  |
| TNM I-II (low-risk) |  |  |  | *Trend* | *0.79* |  |  | *Trend* | *<0.001* |
| 0 | 201 | 71 (3) | 115 | 1.0 (reference) |  | 93 (2) | 20 | 1.0 (reference) |  |
| 1 | 244 | 68 (3) | 152 | 1.08 (0.85-1.38) | 0.52 | 86 (2) | 42 | 1.74 (1.02-2.97) | **0.04** |
| 2 | 54 | 61 (7) | 31 | 1.09 (0.73-1.63) | 0.66 | 66 (7) | 19 | 3.94 (2.10-7.39) | **<0.001** |
| TNM II-III (high-risk) |  |  |  | *Trend* | *0.008* |  |  | *Trend* | *0.001* |
| 0 | 99 | 64 (5) | 61 | 1.0 (reference) |  | 74 (5) | 28 | 1.0 (reference) |  |
| 1 | 180 | 43 (4) | 130 | 1.50 (1.10-2.03) | **0.01** | 54 (4) | 81 | 1.91 (1.24-2.93) | **0.003** |
| 2 | 84 | 40 (5) | 65 | 1.67 (1.18-2.38) | **0.004** | 47 (6) | 45 | 2.34 (1.46-3.76) | **<0.001** |
|  |  |  |  |  |  |  |  |  |  |
| Colon cancer |  |  |  | *Trend* | *0.02* |  |  | *Trend* | *<0.001* |
| 0 | 206 | 72 (3) | 120 | 1.0 (reference) |  | 87 (2) | 33 | 1.0 (reference) |  |
| 1 | 345 | 57 (3) | 232 | 1.29 (1.04-1.61) | **0.02** | 72 (3) | 97 | 1.94 (1.30-2.87) | **0.001** |
| 2 | 99 | 49 (5) | 70 | 1.49 (1.11-2.00) | **0.008** | 57 (5) | 44 | 3.36 (2.14-5.27) | **<0.001** |
| Rectal Cancer |  |  |  | *Trend* | *0.21* |  |  | *Trend* | *<0.001* |
| 0 | 94 | 62 (5) | 56 | 1.0 (reference) |  | 86 (4) | 15 | 1.0 (reference) |  |
| 1 | 79 | 59 (6) | 50 | 1.13 (0.77-1.65) | 0.54 | 71 (5) | 26 | 2.23 (1.18-4.20) | **0.014** |
| 2 | 39 | 46 (8) | 26 | 1.51 (0.95-2.41) | 0.08 | 50 (8) | 20 | 4.07 (2.08-7.96) | **0.001** |

Abbreviation: OS, Overall survival; CSS, Cancer-specific survival

**Supplementary Table S3. GMS and recurrence location (*n*=833).**

| Recurrence location |  | GMS | | | | | | |
| --- | --- | --- | --- | --- | --- | --- | --- | --- |
|  | *N* | **0** (n=293)  *N* (%)^a^ | | **1** (n=424)  *N* (%) | | **2** (n=138)  *N* (%) | | *Pearson X^2^* |
|  |  |  |  |  |  |  |  |  |
| None | 630 | 250 | (85) | 304 | (74) | 76 | (59) | **<0.001** |
| Local | 35 | 8 | (3) | 10 | (2) | 17 | (13) |  |
| Distant | 168 | 35 | (12) | 97 | (24) | 36 | (28) |  |
|  |  |  |  |  |  |  |  |  |

^a^total percentage may not equal 100 as rounded to nearest whole number

**Supplementary Table S4. Univariate RFS for immune cell densities per tumour location and associations between GMS and CD3, CD8 and composite CD3/CD8 score (*N*=208).**

| Group  GMS category |  | Relapse-free Survival | | | | GMS | | | | | | |  |  |
| --- | --- | --- | --- | --- | --- | --- | --- | --- | --- | --- | --- | --- | --- | --- |
|  | *N* | 5-year RFS  (%; SE) | Events (*N*=67) | HR (95% CI) | *P* | **0** (n=74)  *N* (%)^a^ | | **1** (n=96)  *N* (%) | | **2** (n=38)  *N* (%) | | *Pearson X^2^* | |  |
| GMS |  |  |  | *Trend* | *<0.001* |  |  |  |  |  |  |  | |  |
| 0 | 74 | 81 (5) | 16 | 0.38 (0.33-0.67) | **0.001** | – | – | – | – | – | – |  | | |
| 1 | 96 | 72 (5) | 29 | 0.27 (0.14-0.51) | **<0.001** | – | – | – | – | – | – |  | | |
| 2 | 38 | 42 (8) | 22 | 1.0 (reference) |  | – | – | – | – | – | – |  | | |
|  |  |  |  |  |  |  |  |  |  |  |  |  | | |
| CD3 Invasive margin |  |  |  | *Trend* | *<0.001* |  |  |  |  |  |  |  | | |
| Low | 117 | 59 (5) | 49 | 1.0 (reference) |  | 17 | (23) | 75 | (78) | 25 | (66) | **<0.001** | | |
| High | 91 | 84 (4) | 18 | 0.38 (0.22-0.65) | **<0.001** | 57 | (77) | 21 | (22) | 13 | (34) |  | | |
|  |  |  |  |  |  |  |  |  |  |  |  |  | | |
| CD3 Stroma |  |  |  | *Trend* | *<0.001* |  |  |  |  |  |  |  | | |
| Low | 106 | 57 (5) | 48 | 1.0 (reference) |  | 25 | (34) | 57 | (59) | 24 | (63) | **<0.001** | | |
| High | 102 | 83 (4) | 19 | 0.33 (0.19-0.56) | **<0.001** | 49 | (66) | 39 | (41) | 14 | (37) |  | | |
|  |  |  |  |  |  |  |  |  |  |  |  |  | | |
| CD3 Cancer nests |  |  |  | *Trend* | *<0.001* |  |  |  |  |  |  |  | | |
| Low | 139 | 60 (4) | 60 | 1.0 (reference) |  | 33 | (45) | 71 | (74) | 35 | (92) | **<0.001** | | |
| High | 69 | 90 (4) | 7 | 0.18 (0.08-0.39) | **<0.001** | 41 | (55) | 25 | (26) | 3 | (8) |  | | |
|  |  |  |  |  |  |  |  |  |  |  |  |  | | |
| CD8 Invasive margin |  |  |  | *Trend* | *0.001* |  |  |  |  |  |  |  | | |
| Low | 122 | 62 (5) | 49 | 1.0 (reference) |  | 28 | (38) | 69 | (72) | 25 | (66) | **<0.001** | | |
| High | 86 | 82 (4) | 18 | 0.43 (0.25-0.74) | **0.002** | 46 | (62) | 27 | (28) | 13 | (34) |  | | |
|  |  |  |  |  |  |  |  |  |  |  |  |  | | |
| CD8 Stroma |  |  |  | *Trend* | *0.001* |  |  |  |  |  |  |  | | |
| Low | 157 | 64 (4) | 60 | 1.0 (reference) |  | 45 | (61) | 82 | (85) | 30 | (79) | **0.006** | | |
| High | 51 | 88 (5) | 7 | 0.30 (0.14-0.66) | **0.003** | 29 | (39) | 14 | (15) | 8 | (21) |  | | |
|  |  |  |  |  |  |  |  |  |  |  |  |  | | |
| CD8 Cancer nests |  |  |  | *Trend* | *<0.001* |  |  |  |  |  |  |  | | |
| Low | 149 | 61 (4) | 61 | 1.0 (reference) |  | 42 | (57) | 72 | (75) | 35 | (92) | **<0.001** | | |
| High | 59 | 93 (4) | 6 | 0.19 (0.08-0.45) | **<0.001** | 32 | (43) | 24 | (25) | 3 | (8) |  | | |
|  |  |  |  |  |  |  |  |  |  |  |  |  | | |
| Composite CD3/CD8 score |  |  |  | *Trend* | *<0.001* |  |  |  |  |  |  |  | | |
| 0 | 72 | 55 (6) | 33 | 1.0 (reference) |  | 10 | (14) | 48 | (50) | 14 | (37) | **<0.001** | | |
| 1 | 36 | 55 (9) | 16 | 0.96 (0.53-1.74) | 0.88 | 6 | (8) | 20 | (21) | 10 | (26) |  | | |
| 2 | 34 | 82 (7) | 8 | 0.43 (0.20-0.93) | **0.03** | 17 | (23) | 11 | (12) | 6 | (16) |  | | |
| 3 | 38 | 79 (7) | 10 | 0.43 (0.21-0.88) | **0.02** | 23 | (31) | 9 | (9) | 6 | (16) |  | | |
| 4 | 28 | NA | 0 | NA | NA | 18 | (24) | 8 | (8) | 2 | (5) |  | | |
|  |  |  |  |  |  |  |  |  |  |  |  |  | | |

Abbreviation: RFS, Relapse-free survival; HR, hazard ratio; CI, confidence interval; NA, not applicable, incalculable as no terminal events in this category

^a^total percentage may not equal 100 as rounded to nearest whole number

**Supplementary Table S5. Interactions between GMS and chemotherapy Type or duration (*N*=2912).**

|  | GMS category | | | *Interaction P* |
| --- | --- | --- | --- | --- |
|  | **0** (*N)* | **1** (*N)* | **2** *(N)* |  |
| **Full Cohort (n=2912)** | | | | |
| Chemotherapy Type | 383 | 1867 | 663 | ***0.013*** |
| FOLFOX | 120 | 526 | 200 |  |
| CAPOX | 263 | 1341 | 463 |  |
| Chemotherapy Duration | 383 | 1867 | 663 | *0.64* |
| **Lower Risk Stage III Patients (T1-3/N1; n=1284)** | | | | |
| Chemotherapy Type | 202 | 861 | 221 | ***0.005*** |
| Chemotherapy Duration |  |  |  | *0.82* |
| **Higher Risk Stage III Patients (T4 and/or N2; n=1073)** | | | | |
| Chemotherapy Type | 102 | 643 | 327 | *0.61* |
| Chemotherapy Duration |  |  |  | *0.84* |

**Supplementary Table S6. Association between chemotherapy type and duration in GMS 0 subgroup (*N*=383).**

|  | Chemotherapy type | | | Pearson *X*^2^ |
| --- | --- | --- | --- | --- |
|  | **FOLFOX** *N* (%) | **CAPOX** *N* (%) |  |  |
| **GMS 0 (n=383)** | | | | |
| Chemotherapy duration |  |  |  | *0.11* |
|  |  |  |  |  |
| **3-months** | 68 (57) | 52 (48) |  |  |
| **6-months** | 52 (43) | 137 (52) |  |  |


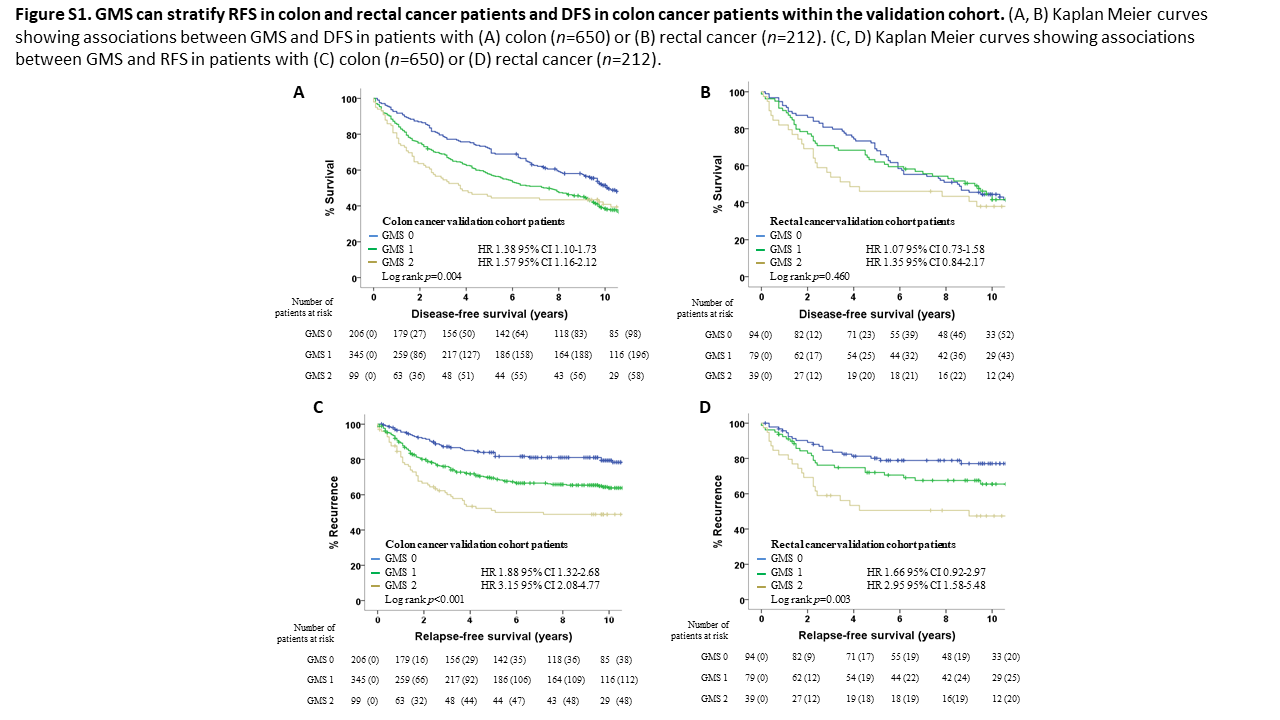


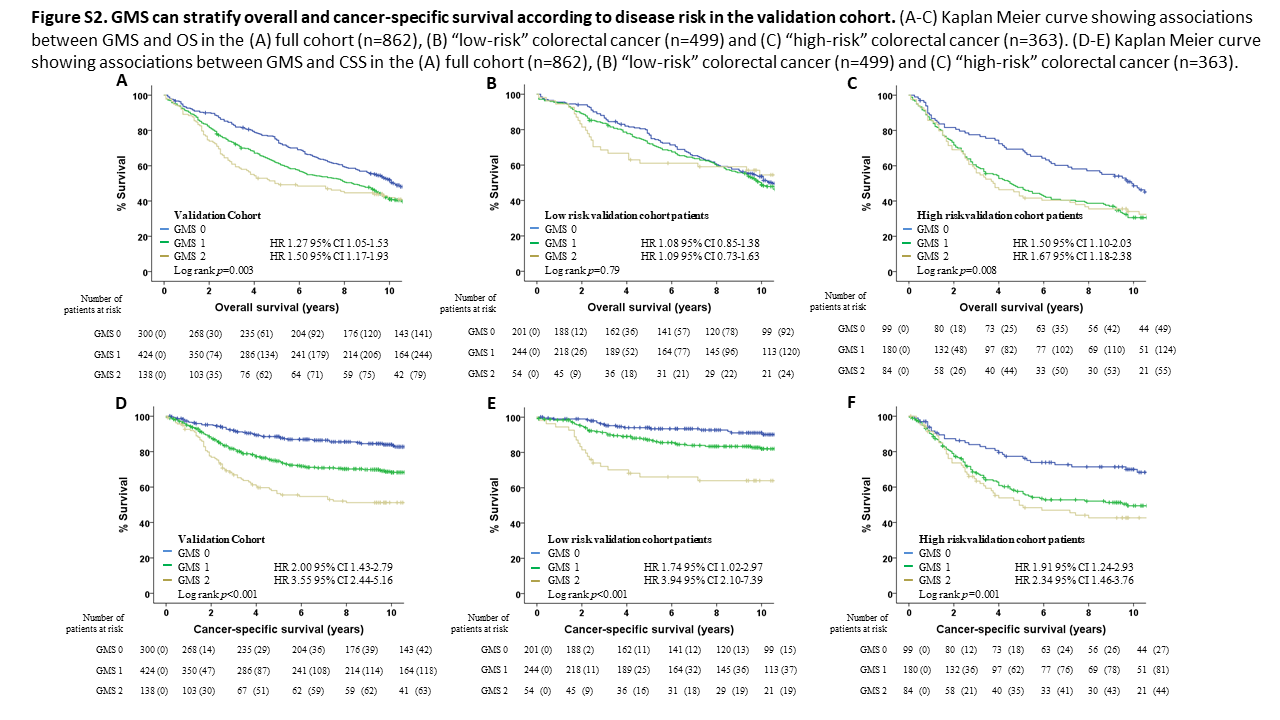


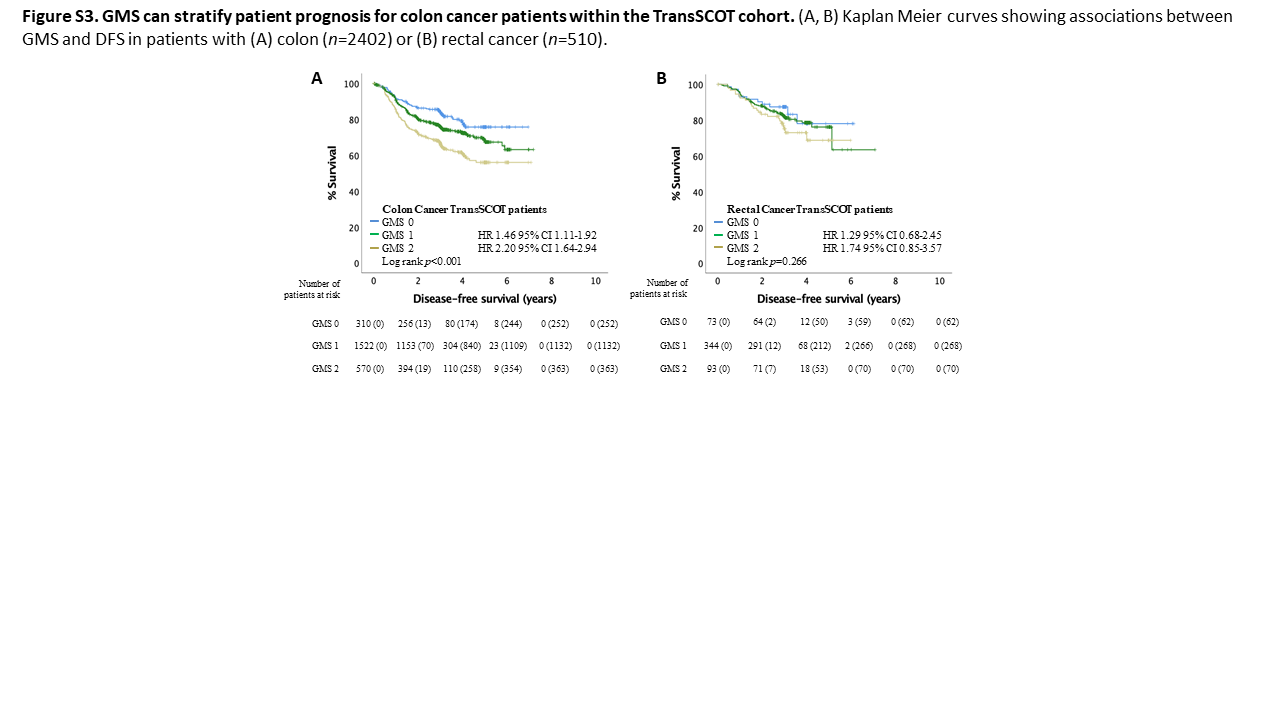

Supplement: Supplementary file 1 — Supplementary data [file 41416_2020_1168_MOESM1_ESM.docx]
